# Correlated network of networks enhances robustness against catastrophic failures

**DOI:** 10.1371/journal.pone.0195539

**Published:** 2018-04-18

**Authors:** Byungjoon Min, Muhua Zheng

**Affiliations:** 1 Department of Physics, Chungbuk National University, Cheongju, Chungbuk 28644, Korea; 2 Levich Institute and Physics Department, City College of New York, New York, New York 10031, United States of America; 3 Department of Physics, East China Normal University, Shanghai, 200062, China; East China Normal University, CHINA

## Abstract

Networks in nature rarely function in isolation but instead interact with one another with a form of a network of networks (NoN). A network of networks with interdependency between distinct networks contains instability of abrupt collapse related to the global rule of activation. As a remedy of the collapse instability, here we investigate a model of correlated NoN. We find that the collapse instability can be removed when hubs provide the majority of interconnections and interconnections are convergent between hubs. Thus, our study identifies a stable structure of correlated NoN against catastrophic failures. Our result further suggests a plausible way to enhance network robustness by manipulating connection patterns, along with other methods such as controlling the state of node based on a local rule.

## Introduction

Real-world complex systems ranging from critical infrastructure [1–3] and transportation networks [4, 5] to living organisms [6–8] are rarely formed by an isolated network but by a network of networks (NoN) [3, 8–30]. For instance, different kinds of critical infrastructures such as a power grid and the Internet are coupled and interact with one another [1, 2]. In addition, many living systems including brain networks [8, 31] and cellular networks [7] consist of different modules strongly connected and interconnections between them.

Several models of a system of networks have been proposed with the role of interconnections that are links across different networks [3, 9, 30]. Models of NoN may fall into three classes according to the functionality of interconnections: Modular NoN (M-NoN), Catastrophic NoN (C-NoN), and Robust NoN (R-NoN). A primitive model of NoN is Modular NoN in which intraconnections within a network and interconnections between different networks have no difference in function [9]. Since nodes connected by an interconnection do not control each other, this model corresponds to a single modular network with a different density of intraconnections and interconnections.

However, considering distinct nature of intraconnections and interconnections in NoN, a different role for different types of connections may be required. For example, when different networks function interdependently, interconnections should not play the same role as intraconnection but control the state of a connected node in the other networks [3, 30]. And, the state of a node in C-NoN model is determined by the global characteristics of a network [3, 32]. To be specific, a node can be active only if any interconnected nodes in different networks belong to the global giant component. Such global rule results in an extreme instability of a system of networks since a small perturbation can trigger catastrophic collapse.

In order to resolve the conflict between the extreme fragility and robust systems of networks observed widely in reality such as the brain, R-NoN model in which the state of a node is controlled by local property of interconnected nodes have been proposed [29, 30]. For R-NoN, nodes connected by an interconnection still control each other. But, a node in R-NoN model can be active even though interconnected nodes in a different network do not belong to the global giant component. With this modification, R-NoN model becomes robust but still maintains the functionality across different networks.

Beside R-NoN, it is of interest how to produce a more robust C-NoN system because there are some examples to follow the global rule such as a power grid. Catastrophic NoN model involves vulnerability related to the global rule leading to the potential danger of abrupt collapse. Here, we investigate a modified model taking into account a correlation in the connectivity patterns of NoN as a remedy of the collapse instabilities. So far, the majority of research about networks of networks have studied NoN with uncorrelated and one-to-one interconnections [3, 32]. In contrast, a system of coupled networks in reality are composed with one-to-many interconnections and a degree-degree correlation between nodes in distinct networks [4, 8, 33–35]. For instance, for the case of the brain networks, non-trivial patterns of connections have been reported for resting state and in task [8]. Correlated coupling was also observed in several different types of complex systems such as transportation networks [35], social networks [33], and critical infrastructure networks [2, 34].

In this study we find that the collapse instabilities in C-NoN can be removed, and the model becomes stable by introducing correlated NoN. Specifically, we investigate the effect of a degree-degree correlation on network robustness under random removal of nodes by extending a previous analysis [8]. We find that when hubs are major source of outgoing links and the interconnections are convergent between hubs, NoN becomes stable to function properly. Our study provides an optimal design of correlated NoN against an external perturbation and a possible reason for stable functioning of correlated NoN in reality.

## Model and theory

We consider a network of networks composed of two networks, *A* and *B*, with interconnections between the networks, for the sake of simplicity. Each node in NoN can have two different types of links, inlinks and outlinks. Inlink refers connections inside the same network while outlink is connections between nodes in different networks.

Here, we examine two different modes of interactions of out-links [8]: Catastrophic NoN and Modular NoN. C-NoN represents that a node in network *A* operates properly only when one of the reciprocal nodes in network *B* connected by outlinks also functions properly. Thus, a node in network *A* cannot be active when it does not belong to the giant component on network *A* or it loses all connectivity to network *B*. On the other hand, for M-NoN, a node in network *A* can be active if it belongs to the giant component through either inlink or outlink. Thus, even though a node in network *A* is completely decoupled from network *B*, such node can be active as long as it still belongs to the giant component of *A*. Therefore, for M-NoN mode of interactions, there is no cascading failure after the initial removal of nodes.

**Fig 1:**
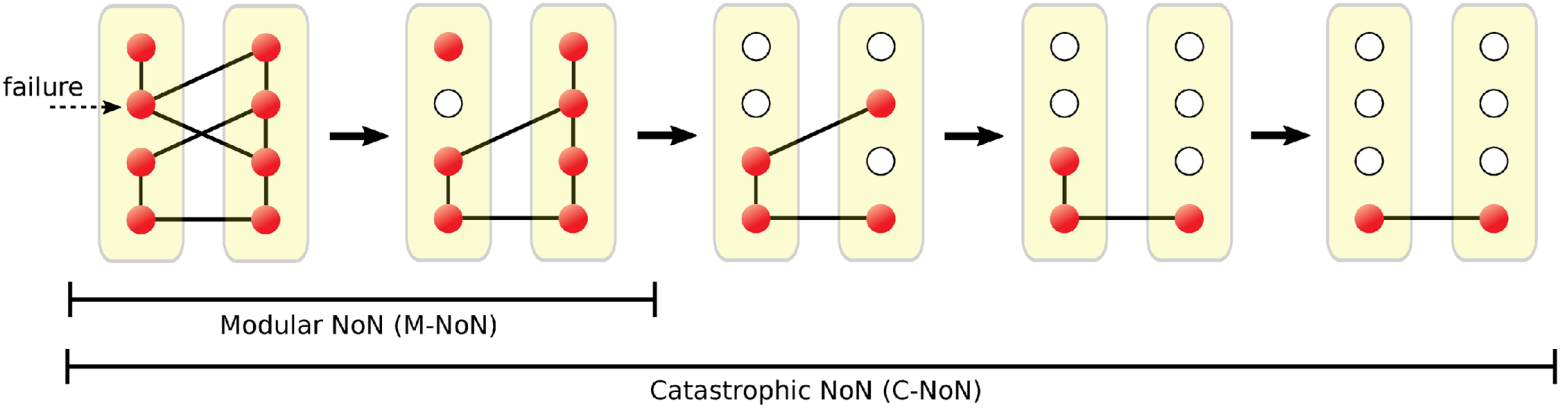
An example of C-NoN and M-NoN. In this example, initially a single node is removed by an external perturbation. For M-NoN, this node and all of its links are removed. For C-NoN, we further remove nodes and their connections if they do not have any interconnections. These removing processes proceed iteratively until there are no more nodes to be removed.

An example of C-NoN and M-NoN is depicted in Fig 1. For M-NoN model, a fraction of nodes are targeted to be removed. Then all targeted nodes and their connections are removed from the original network. Finally we identify the largest connected component linked by either intraconnections or interconnections. For C-NoN model, after the removal of initially targeted nodes, we further remove nodes that do not have any interconnections. In addition, we remove all nodes that do not belong to the largest connected component. So, we remove iteratively nodes that do not belong to the giant component or do not have any interconnections. These removal processes lead to cascading failure.

In order to assess the robustness of a system against random removal, we measure the size of giant component after initial node removal. We also identify the percolation threshold *p*_*c*_ at which the giant component disappears, to measure stability of NoN. NoN with low threshold corresponds to stable structures because many nodes need to be removed to break it down, whereas high percolation threshold represents vulnerable structures.

### Catastrophic network of networks

In this section, we introduce a theory for C-NoN mode of interactions to find the size of giant component and percolation threshold. Initially, all nodes in both networks *A* and *B* are active. A fraction *p*_*A*_ and *p*_*B*_ of nodes randomly chosen are removed from the networks *A* and *B*, respectively. Then, a node is active only if it belongs to the giant component in its network via in-links and at the same time connects to the giant component on the other network via one of its out-links. Nodes that do not satisfy the survival condition are removed from NoN iteratively. Note that nodes that do not have any out-links at the beginning can be active as long as they remain to connect with the giant component via in-links.

To obtain the percolation threshold *p*_*c*_, we introduce a joint degree distributions of indegree and outdegree as $P(\vec{k})$ where $\vec{k}=\left(k_{in}^{A},k_{in}^{B},k_{out}^{A},k_{out}^{B}\right)$. We also introduce a conditional degree distribution for a pair of connected nodes in different networks to take into account a degree-degree correlation, $P_{AB}\left(k_{in}^{A}|k_{in}^{B}\right)$ and $P_{BA}\left(k_{in}^{B}|k_{in}^{A}\right)$. Next, we develop a theoretical framework for the robustness of NoN on a locally tree-like structure with an arbitrary joint degree distribution and a conditional degree distribution [8].

We define *u*_*A*_ and *u*_*B*_ respectively as the probability that a node in networks *A* and *B* reached by a randomly chosen in-link does not belong to a mutually connected giant component. *u*_*A*_ and *u*_*B*_ can be expressed by the following self-consistency equation

$$\begin{array}{ccc} 1-u_{i}= & p_{i} & [\sum_{\vec{k}}\frac{k_{in}^{i}P\left(\vec{k}\right)}{\left\langle k_{in}^{i}\right\rangle }\left(1-u_{i}^{k_{in}^{i}-1}\right)\left(\delta_{k_{out}^{i},0}+1-w_{k_{in}^{i}}^{k_{out}^{i}}\right)], \end{array}$$ (1)

where *i* ∈ {*A*, *B*} and *δ*_*i*,*j*_ is the Kronecker delta. Here, $w_{k_{in}^{i}}$ is the probability that a node reached by a randomly chosen out-link from a node in network *i* with indegree $k_{in}^{i}$ does not belong to the giant component of the opposite network. The first term $\left(1-u_{i}^{k_{in}^{i}-1}\right)$ represents the probability that a node with $k_{in}^{i}$ belongs to the giant component in network *i*, and the second term represents that the probability that a node with $k_{in}^{i}$ connects with the giant component of the opposite network through an outlink. By the term $\delta_{k_{out}^{i},0}$ in Eq (1), a node without out-links ($k_{out}^{i}=0$) can be treated differently with other nodes ($k_{out}^{i}\neq 0$). Then, the probability $w_{k_{in}^{i}}$ can be expressed as

$$\begin{array}{c} 1-w_{k_{in}^{i}}=p_{i}\left[1-\sum_{k_{in}^{i}}P\left(k_{in}^{j}|k_{in}^{i}\right)u_{j}^{k_{in}^{j}}\right]. \end{array}$$ (2)

Obtaining *u*_*i*_ and $w_{k_{in}^{i}}$ by solving these equations, the size *G*_*i*_ of the mutually connected giant component of C-NoN is given by

$$\begin{array}{c} G_{i}=p_{i}\left[\sum_{\vec{k}}P\left(\vec{k}\right)\left(1-u_{i}^{k_{in}^{i}}\right)\left(\delta_{k_{out}^{i},0}+1-w_{k_{in}^{i}}^{k_{out}^{i}}\right)\right]. \end{array}$$ (3)

### Modular network of networks

For M-NoN, a node can survive if it belongs to the giant component a whole network. Given degree distributions, the probability *ν*_*i*_ that a node reached by a randomly chosen inlink of network *i* does not belong to the giant component of M-NoN is given by

$$\begin{array}{c} 1-\nu_{i}=p_{i}\left[\sum_{\vec{k}}\frac{k_{in}^{i}P\left(\vec{k}\right)}{\left\langle k_{in}^{i}\right\rangle }\left(1-\nu_{i}^{k_{in}^{i}-1}\mu_{k_{in}^{i}}^{k_{out}^{i}}\right)\right]. \end{array}$$ (4)

Here, $\mu_{k_{in}^{i}}$ is the probability that a node reached by a randomly chosen outlink from a node in network *i* with indegree $k_{in}^{i}$ does not belong to the giant component of the opposite network. And, the probability $\mu_{k_{in}^{i}}$ can be obtained by following,

$$\begin{array}{c} 1-\mu_{k_{in}^{i}}=p_{i}\left[1-\sum_{k_{in}^{i}}P\left(k_{in}^{j}|k_{in}^{i}\right)\nu_{j}^{k_{in}^{j}}\right]. \end{array}$$ (5)

For M-NoN, a node in network *i* can survive if it belongs to the giant component in network *i* or the giant component in a different network by an interconnection. Once we obtain *ν*_*i*_ and $\mu_{k_{in}^{i}}$, the size *G*_*i*_ of the giant component of M-NoN is

$$\begin{array}{c} G_{i}=p_{i}\left[\sum_{\vec{k}}P\left(\vec{k}\right)\left(1-\nu_{i}^{k_{in}^{i}}\mu_{k_{in}^{i}}^{k_{out}^{i}}\right)\right]. \end{array}$$ (6)

### Correlation in network of networks

**Fig 2:**
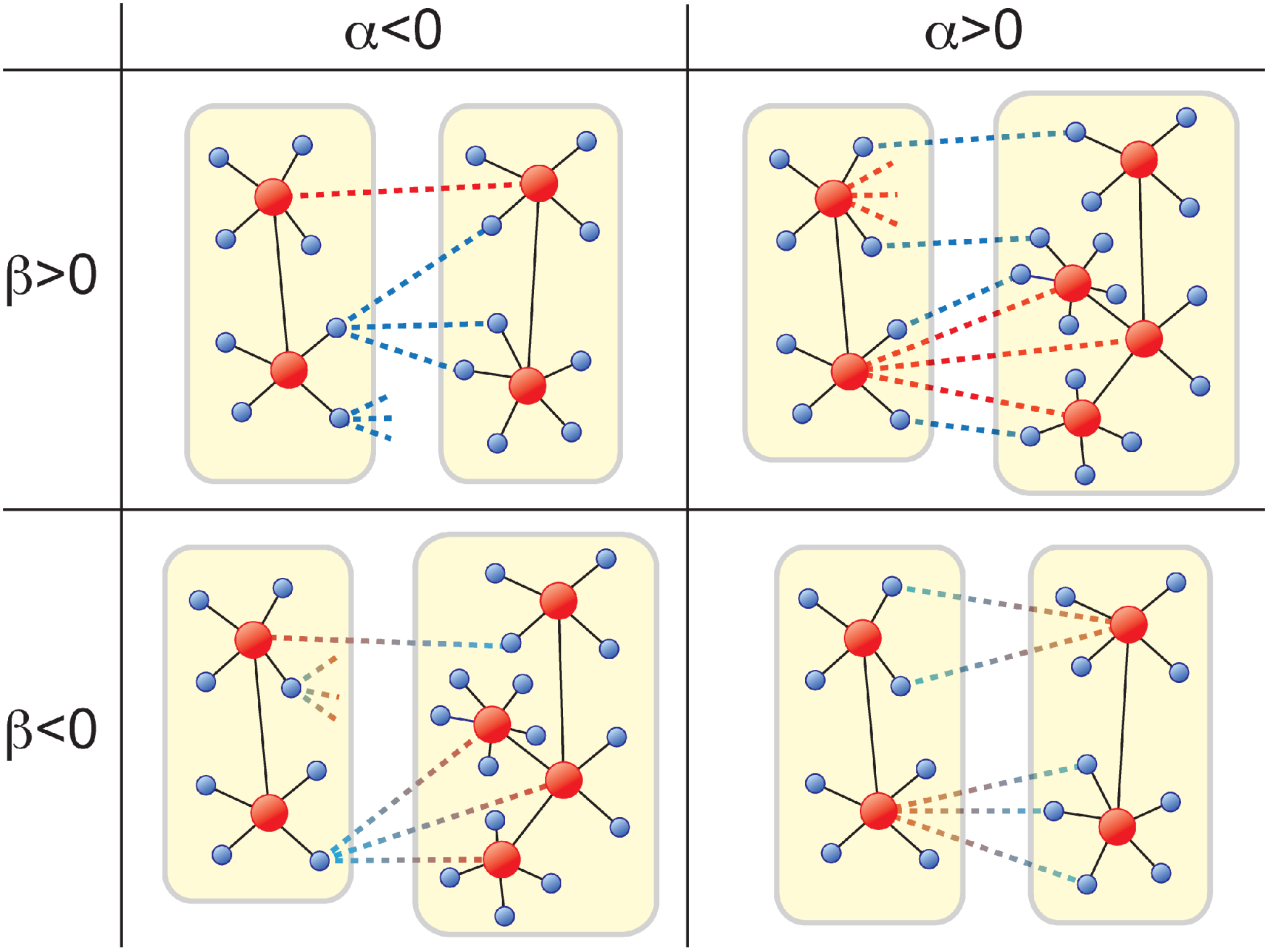
Diagram of a correlated network of networks according to parameters *α* and *β*. Hubs (red nodes) and non-hubs nodes (blue nodes) can have inlinks (solid lines) and outlinks (dotted lines). When *α* > 0, hubs are more likely to have many outlinks whereas when *α* < 0, non-hub nodes are more likely to have outlinks. When *β* > 0, hubs prefer to connect with other hubs in a different network but when *β* < 0, hubs in one network prefer to connect to non-hub nodes in a different network.

In real-world complex systems, NoN are not made randomly but with a certain degree-degree correlation. Correlated coupling is observed in several different kinds of complex systems such as transportation networks [35], social networks [33], and critical infrastructure networks [2, 34], and crucial for structural and dynamical properties of networks [36–38]. For instance, functional brain networks of the human show a peculiar correlation pattern [8]. In this paper, we consider a degree-degree correlation using two scaling parameters, *α* and *β* (Fig 2) as observed in functional networks of the human brain [8]. The parameter *α* is defined as

$$\begin{array}{c} k_{out}∼k_{in}^{\alpha }. \end{array}$$ (7)

Thus, for *α* > 0 hubs of each network also have many outlinks, whereas for *α* < 0 nodes with low degree have many outlinks (Fig 2). The other parameter *β* is defined as

$$\begin{array}{c} k_{in}^{nn}∼k_{in}^{\beta }, \end{array}$$ (8)

where $k_{in}^{nn}$ is the average indegree of the nearest neighbors in the other network. Therefore, *β* quantifies indegree-indegree correlation between two connected nodes by interconnections. For *β* > 0, hubs connect with other hubs in the different network. Instead for *β* < 0, hubs in a network connect with nodes with less degree in the other network (Fig 2). Note that uncorrelated NoN corresponds to *α* = 0 and *β* = 0.

## Results

### Effect of the density of out-links

We first examine the robustness of NoN by changing the density of links in order to check the effect of outlinks. As an instructive example, we consider a coupled Erdös-Rényi (ER) network. For ER NoN with no degree correlation, a joint degree distribution can be factorized as $P\left(\vec{k}\right)=P_{in}\left({\vec{k}}_{in}\right)P_{out}\left({\vec{k}}_{out}\right)$ and a conditional degree distribution can be simply expressed as $P\left(k_{in}^{j}|k_{in}^{i}\right)=P_{in}\left(k_{in}\right)$. We assume that two networks have the same average in-degree, $\langle k_{in}^{A}\rangle =\langle k_{in}^{B}\rangle =\langle k_{in}\rangle$, and the fraction of removed nodes are the same for both networks, *p*_*A*_ = *p*_*B*_ = *p*. Then, Eqs (1) and (2) can be simply reduced into a single equation:

$$\begin{array}{c} u=1-p\left[1-e^{\left\langle k_{in}\right\rangle \left(u-1\right)}\right]\left[e^{-\langle k_{out}\rangle }+1-e^{p\left\langle k_{out}\right\rangle \left(e^{\left\langle k_{in}\right\rangle \left(u-1\right)}-1\right)}\right]. \end{array}$$ (9)

where 〈*k*_*out*_〉 is the average outdegree. Once we define the function

$$\begin{array}{c} f\left(u\right)=u-1+p\left[1-e^{\left\langle k_{in}\right\rangle \left(u-1\right)}\right]\left[e^{-\langle k_{out}\rangle }+1-e^{p\left\langle k_{out}\right\rangle \left(e^{\left\langle k_{in}\right\rangle \left(u-1\right)}-1\right)}\right], \end{array}$$ (10)

one can obtain the percolation threshold *p*_*c*_ by imposing the conditions *f*(*u*) = *f*′(*u*) = 0. In addition, a tricritical line (〈*k*_*in*_〉, 〈*k*_*out*_〉, *p*) between continuous and discontinuous transitions can be computed by the conditions *f*(*u*) = *f*′(*u*) = *f*′′(*u*) = 0.

For M-NoN, the self-consistency equation is similarly given by

$$\begin{array}{ccc} 1-\nu & = & p[1-e^{\left\langle k_{in}\right\rangle \left(\nu -1\right)}e^{\left\langle k_{out}\right\rangle p\left(e^{\left\langle k_{in}\right\rangle \left(\nu -1\right)}-1\right)}]. \end{array}$$ (11)

Then, one can obtain the percolation threshold with the conditions *g*(*ν*) = *g*′(*ν*) = 0, if we define

$$\begin{array}{c} g\left(\nu \right)=\nu -1+p\left[1-e^{\left\langle k_{in}\right\rangle \left(\nu -1\right)}e^{\left\langle k_{out}\right\rangle p\left(e^{\left\langle k_{in}\right\rangle \left(\nu -1\right)}-1\right)}\right]. \end{array}$$ (12)

Note that the percolation transition of M-NoN is always second-order and hence a tricritical point does not exist.

**Fig 3:**
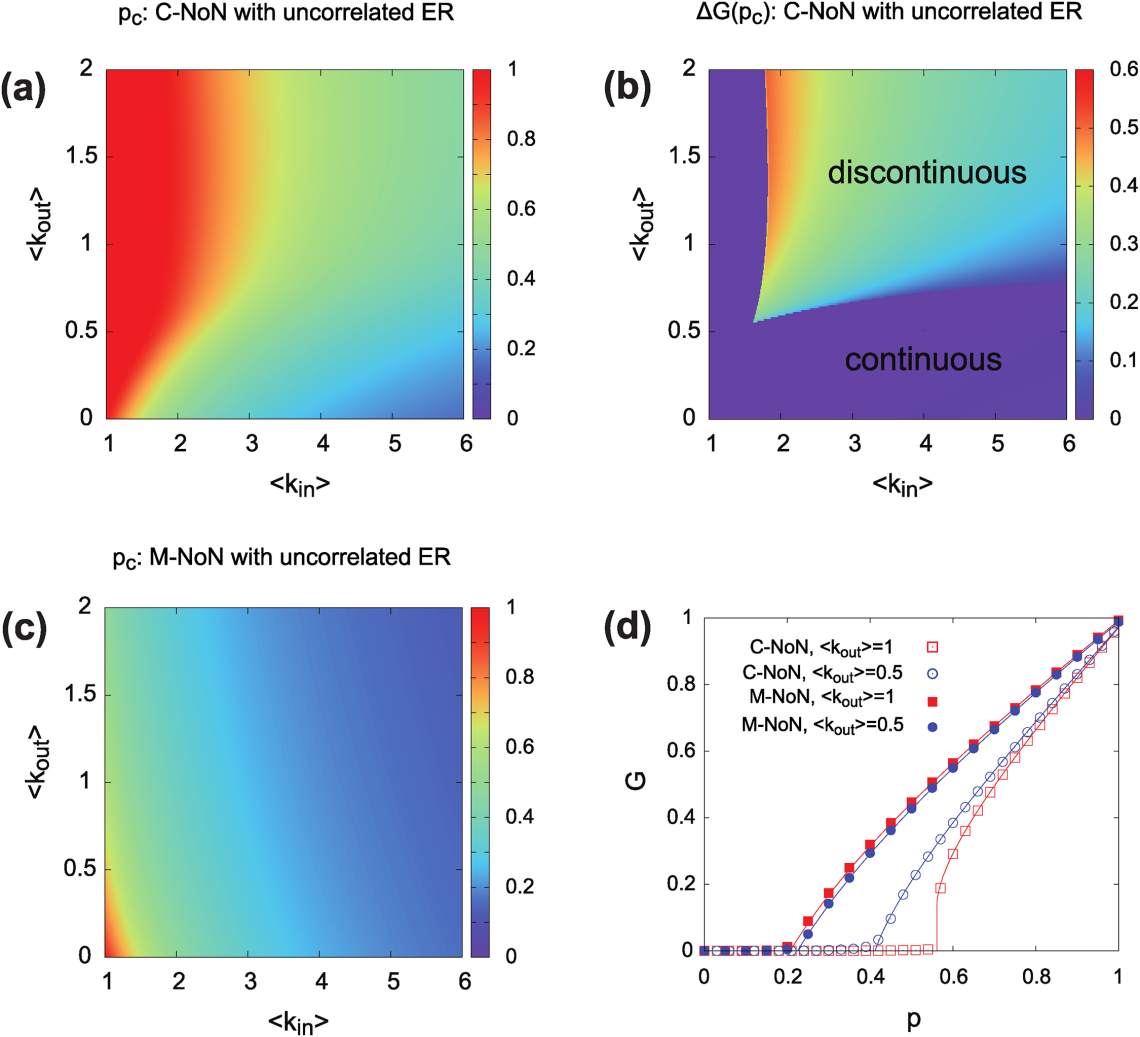
(a) Percolation threshold *p*_*c*_ of C-NoN for two coupled ER networks with no correlation predicted by theory. For high 〈*k*_*out*_〉 and low 〈*k*_*in*_〉, NoN is stable to maintain mutual connectivity under the random removal of nodes. (b) The size of jump at the percolation threshold of C-NoN. The size of jump shows undergoes a second-order phase transition for small 〈*k*_*out*_〉, but the transition becomes discontinuous as 〈*k*_*out*_〉 increases. (c) Percolation threshold *p*_*c*_ of M-NoN for ER NoN with no degree correlation. NoN becomes more stable with increasing either 〈*k*_*in*_〉 or 〈*k*_*out*_〉. (d) The size of giant component for both C-NoN (open symbol) and M-NoN (filled symbol) modes of interactions as a function *p* of a fraction of removed nodes. Analytic calculation (line) and numerical simulation (symbols) are shown together.

Increasing the density of out-links, NoN with catastrophic interactions becomes getting vulnerable as depicted in Fig 3(a). In addition, the transition between percolating and non percolating phases becomes discontinuous above a tricritical line and the size of discontinuous jump at the transition increases with increasing 〈*k*_*out*_〉 [Fig 3(b)]. For C-NoN, outlinks force interconnected systems to be more vulnerable and prone to abrupt collapse due to cascading failure. On the other hand, inlinks preserve the connectivity and produce more robust structures. In conclusion, NoN with high 〈*k*_*in*_〉 and low 〈*k*_*out*_〉 shows a stable structure for C-NoN.

For M-NoN, however, outlinks play the opposite role. High density of outlinks enhances network robustness by adding a potential detour for connectivity [Fig 3(c)]. Outlinks contribute to maintain the robustness of networks for M-NoN but they can cause the opposite effect for C-NoN. Thus, the optimal design of interconnections between networks is called for maintaining stable functioning for both M-NoN and C-NoN.

### Generating correlated networks of networks

In order to examine the effect of a degree-degree correlation, we first construct NoN with a correlation (*α*, *β*). We construct a network drawn from an indegree distribution *P*_*i*_(*k*_*in*_), by following configuration model. Next, stubs of outgoing links are assigned to each node with the probability proportional to $k_{in}^{\alpha }$. Connecting two nodes in different networks with a relationship $k_{in}^{nn}∼k_{in}^{\beta }$ is non-trivial. We cannot simply assign a set of connections for outlinks from a joint distribution $P(\vec{k})$ since such a set almost certainly fails to satisfy the topological constraint because of the reciprocal relation between $k_{in}^{nn}∼k_{in}^{\beta }$ and $k_{in}^{nn}∼k_{in}^{1/\beta }$, except for *β* = 0 and *β* = 1.

**Fig 4:**
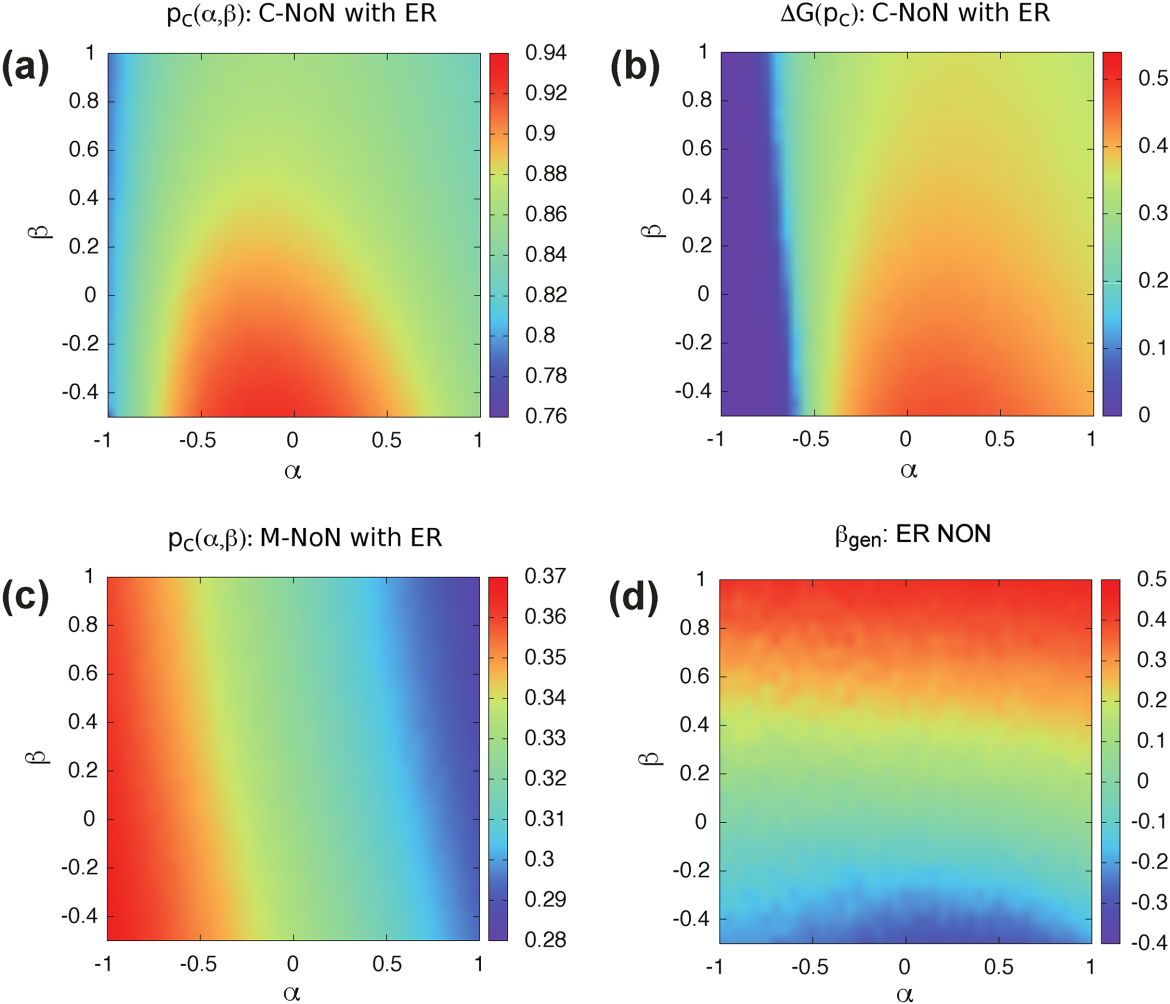
(a) Percolation threshold and (b) size of jump of C-NoN in correlated ER NoN with *N* = 10^4^, 〈*k*_*in*_〉 = 2, and 〈*k*_*out*_〉 = 1 for different *α* and *β*. When *α* ≈ −1 or *α* > 0.5 and *β* > 0, NoN becomes stable against random failure. In contrast, when −0.5 < *α* < 0.5 and *β* < 0, NoN is vulnerable to catastrophic collapse. (c) percolation threshold of M-NoN with correlated ER NoN with the same parameters as C-NoN. High *α* and *β* region is robust against random failure for M-NoN. (d) *β*_*gen*_ observed from realized networks at a given (*α*, *β*). The value *β*_*gen*_ is obtained by a linear regression.

**Fig 5:**
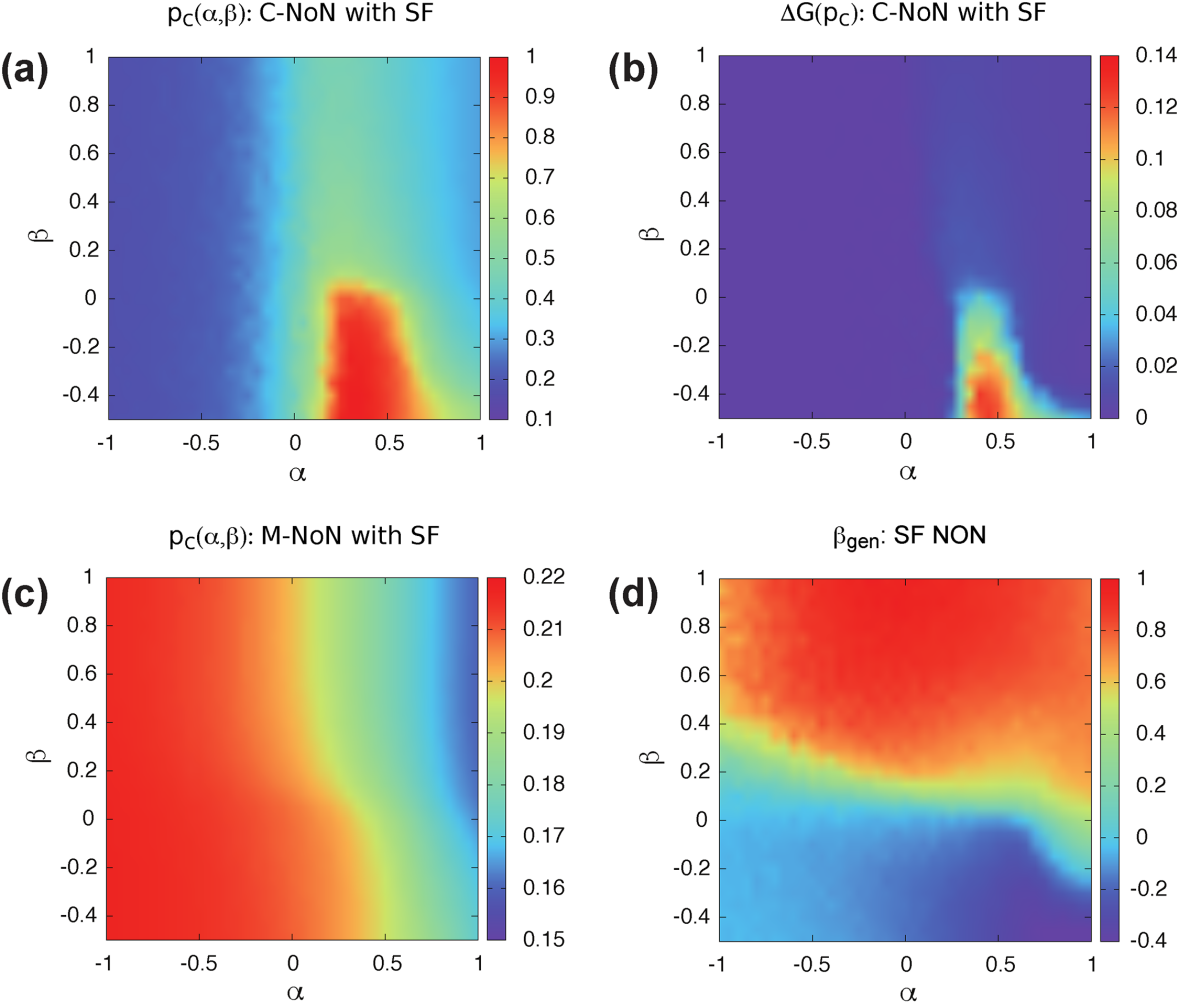
(a) Percolation threshold, (b) size of jump for C-NoN, and (c) percolation threshold for M-NoN with two coupled SF networks with *N* = 10^4^, 〈*k*_*out*_〉 = 1, the degree exponent *γ* = 2.5, and *k*_*max*_ = 100 for different *α* and *β*. High *α* and *β* region is robust against random failure for both C-NoN and M-NoN. When *α* < 0 or *α* > 0.5 and *β* > 0, NoN becomes stable against random failure. In contrast, when −0.5 < *α* < 0.5 and *β* < 0, NoN is vulnerable to catastrophic collapse. (d) *β*_*gen*_ obtained by a linear regression from realized networks at a given (*α*, *β*).

Instead, we use the following way as in [8] to construct NoN with a degree-degree correlation *β*. We choose randomly node *i* in network *A* if it has available outlinks. Next, we connect node *i* with node *j* with degree $k_{in}^{B}$ in network *B* with the probability that follows a Poisson distribution $P(k_{in}^{j})$ with a mean value $\lambda =\langle C_{\beta }k_{in}^{\beta }\rangle$ where $C_{\beta }=k_{max}^{(1-\beta )/2}$. This processes repeat until there are no more out-links left. This algorithm cannot make NoN with exactly corresponding *β* for most sets of (*α*, *β*), but it can guarantee that numerically generated *β*_*gen*_ increases or decreases in a monotonic manner with changing *β* [Figs 4(d) and 5(d)].

### Robustness of correlated networks of networks

To search robust structures of correlated NoN, we generate NoN with the above algorithm and obtain joint and conditional degree distributions from the realized networks with (*α*, *β*). Next, we identify the critical fraction *p*_*c*_ of nodes removal by imposing the condition *G*(*p*_*c*_) = 0, showing network robustness with a given correlation. In order to examine the effect of the correlated structure of NoN, we calculate *p*_*c*_(*α*, *β*) for the both modes of C-NoN and M-NoN with ER networks and scale-free (SF) networks. The small *p*_*c*_(*α*, *β*) represents robust structures against an external perturbation.

For ER NoN, when *α* ≈ −1, low *p*_*c*_ is observed regardless of *β*, indicating stable NoN [Fig 4(a)]. In this region, hubs are isolated in a single network and maintain effectively the giant component. As a result, the extensive size of jump at *p*_*c*_ vanishes [Fig 4(b)]. Another stable region is located at *α* > 0.5 and *β* > 0. High *α* and *β* guarantees that many hub-hub interconnections, so that hubs are more likely protected from cascading failure. When −0.5 < *α* < 0.5 and *β* < 0, a system of networks is highly vulnerable to catastrophic cascading failure. With these parameters, hubs connect to nodes with less degree nodes in the other network, leading to that hubs can be easily attacked by interdependency. For M-NoN, the network robustness enhances with increasing *α* and *β* monotonically [Fig 4(c)]. When *α* > 0 and *β* > 0, both inlinks and outlinks converge toward hubs and the giant component can be preserved with only a few hubs. Therefore, high *α* and *β* region is robust against random failure for M-NoN.

The impact of the correlation is more clear in SF networks because of a key role of hubs with an inhomogeneous degree distribution. When *α* < 0, a networked system is stable (low *p*_*c*_) because hubs are protected from cascading failure for N-NoN [Fig 5(a)]. When *α* > 0.5 and *β* > 0, networks are also stable since hubs are more likely active due to a lot of interconnections between them. However, for intermediate *α* (0 < *α* < 0.5) and divergent interconnections (*β* < 0), hubs are easily exposed to cascading failure since they connect to non-hub nodes in the other network. In this region, C-NoN is fragile to random attack and results in abrupt collapse as shown in Fig 5(b). For M-NoN, a coupled SF network is more vulnerable when *α* < 0 because hubs have only few outlinks as in ER NoN [Fig 5(c)].

In conclusion, the degree-degree correlation in NoN allows us to find a stable structure for functioning of NoN. When hubs have many interconnections (*α* ≈ 1) and hub-hub interconnections are abundant (*β* > 0), NoN can maintain a robust structure for both C-NoN and M-NoN. And, M-NoN is vulnerable when *α* < 0 and C-NoN is at risk of catastrophic collapse when *β* < 0.

## Discussion

We study the robustness of a system of networks with degree-degree correlations and one-to-many interconnections between distinct networks. We investigate the effect of degree-degree correlations on the network robustness with different modes of interconnections. For uncorrelated NoN, outlinks reduce the robustness for C-NoN while they enhance the robustness for M-NoN. However, taking into account the degree correlation, we find stable structures in correlated networks of networks for both C-NoN and M-NoN. Specifically, when hubs provide most interconnections and the interconnections are convergent, networks of networks become more robust for both modes of interconnections. Our study of correlated NoN can shed light on finding the origin of reliable functioning of interconnected networks in reality. In addition, it can provide an economical method of designing robust multilayered systems such as interconnected infrastructures or financial systems. In addition to correlated NoN, robust NoN model which is recently proposed [29, 30] can be another plausible solution of stable functioning of NoN and also allow us to find the core areas in NoN [30, 39–45].
